# Dynamic profile of differentiated thyroid cancer in male and female patients with thyroidectomy during 2000–2013 in China: a retrospective study

**DOI:** 10.1038/s41598-017-14963-z

**Published:** 2017-11-20

**Authors:** Hui-xian Yan, Ping Pang, Fu-lin Wang, Wen Tian, Yu-kun Luo, Wei Huang, Guo-qing Yang, Nan Jin, Li Zang, Jin Du, Jian-ming Ba, Jing-tao Dou, Yi-ming Mu, Zhao-hui Lyu

**Affiliations:** 1 Department of Endocrinology, Chinese PLA General Hospital, Beijing, 100853 China; 2 Beijing Haidian Hospital, Beijing Haidian Section of Peking University Third Hospital, Beijing, 100080 China; 3 Department of Endocrinology, Hainan Branch of PLA General Hospital, Sanya, 572013 Hainan China; 4 Department of Pathology, Chinese PLA General Hospital, Beijing, 100853 China; 5 Department of General Surgery, Chinese PLA General Hospital, Beijing, 100853 China; 6 Department of Ultrasonography, Chinese PLA General Hospital, Beijing, 100853 China

## Abstract

The study aimed to investigate the gender-related differences of disease onset, age distribution, blood type, clinical characteristics, and malignant behaviors of differentiated thyroid carcinoma (DTC) in Chinese patients. A total of 7385 consecutive thyroid cancer patients who underwent thyroidectomy were retrospectively reviewed. 4087 (55.3%) were diagnosed as benign and the other (3298, 44.7%) were as malignant. DTC accounted for 97.6% in the malignant tumor. More single nodules turned out to be DTC in male compared to multiple nodules (46.9% vs. 40.4%, P = 0.004). The proportion increased along with the increase of year during 2000–2013, which was from 7.5% to 68.1% in males and from 16.2% to 66.7% in females. The level of preoperative TSH was significantly higher in patients with DTC compared to the patients with benign (1.97 vs. 1.57 mIU/L, P < 0.001). The proportion of thyroid cancer was dominated in blood type B and the lowest incidence in blood type A in male, the difference was not statistically significant. The results showed that age, nodule number, BMI and serum TSH were the related factors for DTC. More aggressive behaviors of DTC were observed in male patients, and more attention should be focused on the timely diagnosis and treatment of these patients.

## Introduction

Thyroid cancer is a rare malignancy that occurs more frequently in female. The incidence of thyroid cancer has been steadily rising worldwide in recent years^1^. In the United States, the estimated overall incidence of thyroid cancer has been increased by 6.6% annually from 2000 to 2009, ranking the first among all cancers^1^. According to the surveillance of Epidemiology and End Results program, the age-adjusted incidence rate of thyroid cancer has been reported to be about 13.5 per 100,000 people in 2015^2^. The high variability (up to nearly tenfold) in the thyroid cancer incidence was explained by genetic factors, environmental influences, and access to medical care, geographic area and ethnicity.

Differentiated thyroid carcinoma (DTC) is the most common type of thyroid cancer, accounting for as high as 90% of all patients^3^. It originated from thyroid follicular epithelial cells, and mainly includes papillary thyroid carcinoma (PTC), follicular thyroid carcinoma (FTC), and a minority of Hiirthle cell carcinoma or eosinophilic cell carcinoma^3^. More and more studies have been focused on DTC^4–7^, but the gender related characteristics of the disease have not been well established. Moreover, the ABO blood type, as a marker of familial and genetic factors, has been reported to associate with the risk of several malignancies, including gastric, epithelial ovarian and pancreatic cancer^8–10^. However, few studies have been focused on the possible association between ABO blood type and the risk of thyroid cancers in Chinese patients.

Therefore, the study aimed to investigate the gender-related differences of disease onset, age distribution, blood type, clinical characteristics, and malignant behaviors of DTC in Chinese patients.

## Material and Methods

### Patients

This study was approved by the Ethical Committee of the institutional review board of the People Liberation Army General Hospital (Beijing, China) and written informed consent was obtained from all patients. All methods were performed in accordance with the relevant guidelines and regulations.

A total of 7,385 consecutive thyroid cancer patients who underwent thyroidectomy at the Department of General Surgery, PLA General Hospital during January 2000 to January 2013 were retrospectively reviewed. All patients who were diagnosed with thyroid cancer were pathologically confirmed and had complete medical records. The physical examination and laboratory tests were performed. Fine needle aspiration biopsy (FNAB) test was performed only in patients with suspicious US features.

Surgical indications were more than one thyroid nodule confirmed by ultrasound with suspicious US features: 1) Micro calcifications, infiltrative margins, anteroposterior/transversal (AP/TR) diameters ≥1, solitary, and hypoechoic, large goiter with findings suggestive of malignancy or indeterminate/suspicious FNAC finding; 2) With clinical symptoms of hoarseness, dysphagia and compression symptoms associated with nodules; 3) No contraindications for surgical procedure; 4) The informed consent of the patients for surgery.

Laboratory data were obtained from all patients, including thyroid profiles, ABO blood type, and thyroid ultrasonography. The basic information and medical histories of the patients were collected by professional doctors.

### Laboratory tests

Preoperative blood tests were performed one day to one week prior to thyroidectomy with the patients in a fasting state. Laboratory data available were ABO blood type and thyroid profile, consisting of serum Thyroid Stimulating Hormone (TSH), thyroid peroxidase antibody (TPO-Ab) and thyroglobulin antibody (TG-Ab). Laboratory data were measured with the same automated immune chemiluminescent assay (ICMA, Abbott, Abbott Park, IL, USA) at the laboratory of our hospital. The sensitivity of TSH assay was 0.01 mIU/L. Serum TPO-Ab of >60 U/L and/or TG-Ab of >60 U/L were considered as high serum autoantibody values.

Thyroid ultrasonography was performed using a 5- to 17-MHz linear array transducer (LOGIQ 9, GE Healthcare, Milwaukee, WI, or iU22, Philips Medical Systems, Bothell, WA). The images were reviewed at the Picture Archiving and Communication System (PACS, GE Medical System, Milwaukee, WI, USA) by two experienced nuclear medicine physicians that blinded to the original diagnosis of the disease. Data of thyroid nodule size, number, echo pattern characteristics, calcification and blood flow were recorded.

### Pathology

The postoperative histological results were available for the entire cohort of the patients. The results were reviewed by an experienced pathologist to confirm the histology of the primary lesion (benign or malignant nodules) and the pathological types of malignant nodules. In patients with multifocal thyroid cancer, the largest tumor diameters of the nodules were reported. Status of extra thyroidal invasion, lymph node metastasis, and distant metastasis were recorded and the tumors were staged according to the staging system of the American Joint Committee on Cancer.

### Statistical analysis

Body mass index (BMI) was calculated by the weight (kg) divided by the square of height (m). Obesity was defined as BMI ≥ 28 kg/m^2^ and overweight was defined as BMI ≥ 24 kg/m^2^ and BMI < 28 kg/m^2^ recommended by the Working Group on Obesity in China^11^. Serum TSH values were classified as follows: ≤0.3 mIU/L, 0.3–1.0 mIU/L, 1.0–1.9 mIU/L, 1.9–4.8 mIU/L and >4.8 mIU/L, based on cutoff values predetermined in a previous population study^12^. Categorical data were presented as count and percentage. Continuous variables were presented as mean and standarad deviation (SD), or median with the interquartile range (IQR) due to their non-normal distributions. For continuous data, differences between females and males were compared using the nonparametric Mann-Whitney test. Frequency distributions were compared with the χ^2^ test. Multiple logistic regression model was used to evaluate the related factor. All statistical analysis were two-sided and considered significant when p < 0.05. Statistical analyses were performed using the statistics software, SPSS 19.0 (SPSS Inc., Chicago, IL, USA).

### Statement of Ethics

The appropriate ethics review board approved the study design.

## Results

### Characteristics of patients

In total 7,385 patients were recruited in the study, which consisted of 5,246 (71.0%) females and 2,139 (29.0%) males. The mean ± SD age of the patients was 47 ± 12 years in females and 48 ± 13 years in males, respectively. Among them, 4,087 (55.3%) were diagnosed as benign and the other (3,298, 44.7%) were as malignant. DTC accounted for 97.6% (3,218/3,298) in the malignant tumor. 80 patients were non-DTC follicular-type papillary carcinoma nodules (1.1%), which were further consisted of 37 medullary carcinoma nodules, 34 follicular carcinoma nodules, 14 anaplastic carcinoma nodules, 7 malignant lymphoma nodules, 1 metastatic carcinoma, and 21 Hürthle cell adenomas (HCA).

The patients with benign tumor had an older age, larger nodule size and lower TSH level than those with DTC whether in male or in female (all P < 0.05) in Table 1. More single nodules turned out to be DTC in male compared to multiple nodules (46.9% vs. 40.4%, P = 0.004). While no significantly difference in female was observed (44.4 vs. 44.4%, P = 0.922).

**Table 1:** The characteristics of patients diagnosed as benign or DTC in the study.

Factor	Total			Male			Female		
	DTC (3218)	Benign (4087)	P	DTC (908)	Benign (1200)	P	DTC (2310)	Benign (2887)	P
Age, mean ± SD, years	44 ± 12	49 ± 12	<0.001	44 ± 12	52 ± 12	<0.001	44 ± 11	48 ± 12	<0.001
Nodule size, mean ± SD, cm	1.4 ± 1.2	3.0 ± 1.7	<0.001	1.5 ± 1.2	3.2 ± 1.8	<0.001	1.3 ± 1.1	2.9 ± 1.7	<0.001
Body mass index, kg/m^2^	24.6 ± 3.5	24.5 ± 3.4	0.146	26.4 ± 3.4	25.6 ± 3.1	<0.001	23.9 ± 3.3	24.0 ± 3.4	0.275
Nodule type*			0.124			0.004			0.922
Single (2423)	1095	1328		369	418		726	910	
Multiple (4846)	2098	2748		529	780		1569	1968	
TSH, median (IQR), mIU/l	1.97	1.57	<0.001	1.66	1.44	0.001	2.08	1.62	<0.001
	(1.21–3.05)	(0.89–2.55)		(1.03–2.56)	(0.9–2.19)		(1.30–3.26)	(0.89–2.76)	

Note: TSH levels are presented as median (interquartile range); DTC, differentiated thyroid cancer; *there were 2423 patients with solitary and 4846 with multiple.

**Figure 1:**
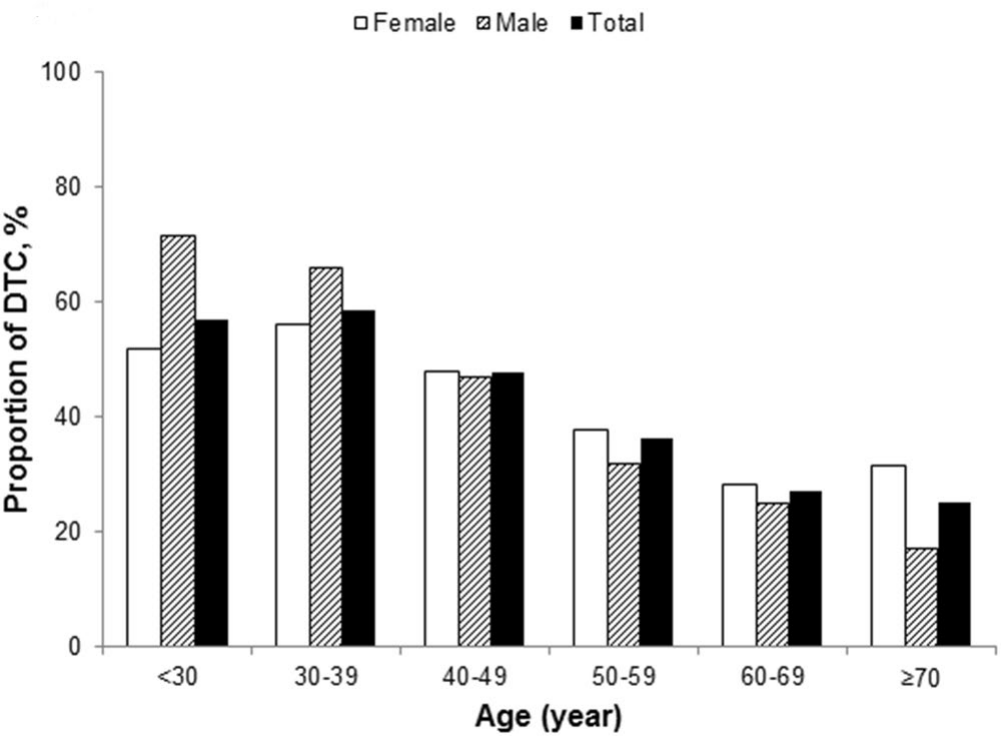
The distribution of differentiated thyroid cancers in different age groups.

The peak of DTC proportion in thyroid nodules was at the group of 30–39 years in female and of younger than 30 years in male among the patients with thyroidectomy. And then the proportion of DTC decreased along with the increase of age (Fig. 1). Compared with the characteristics of male and female thyroid cancer patients, we found that there were significant differences in nodule size, BMI, nodular type and TSH between the two groups (Table 2, all P < 0.001). The nodule size and BMI of the male group were significantly higher than those of the female group (1.5 vs. 1.3; 26.4 vs. 23.9). For the composition of the nodular type, the proportion of single in the male group accounted for 41.1%, while the female group was 31.6% (P < 0.001). The median TSH in the male group was 1.66, which was significantly lower than that in the female group (2.08).

**Table 2:** Comparison of characteristics of thyroid cancer in men and women.

Factor	Male	Female	P value
Age, mean ± SD, years	44 ± 12	44 ± 11	0.609
Nodule size, mean ± SD, cm	1.5 ± 1.2	1.3 ± 1.1	<0.001
Body mass index, kg/m^2^	26.4 ± 3.4	23.9 ± 3.3	<0.001
Nodule type*			<0.001
Single (1095)	369(41.1%)	726(31.6%)	
Multiple (2098)	529(58.9%)	1569(68.45)	
TSH, median (IQR), mIU/l	1.66(1.03–2.56)	2.08(1.30–3.26)	<0.001

Note: TSH levels are presented as median (interquartile range); DTC, differentiated thyroid cancer; *there were 1095 patients with solitary and 2098 with multiple.

### Years

**Figure 2:**
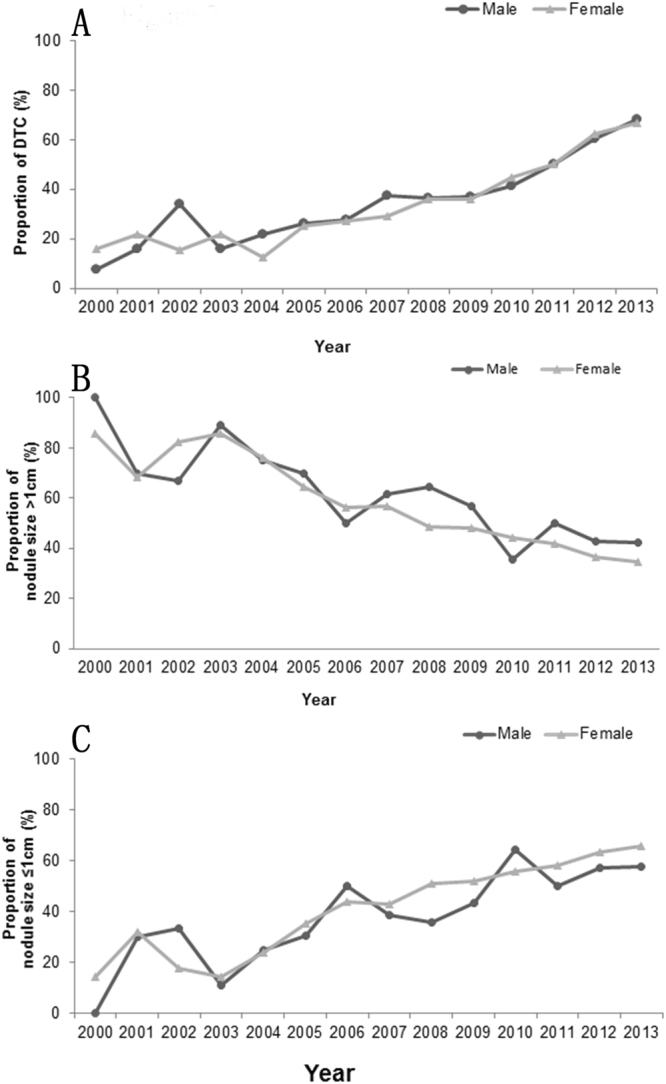
The dynamic profile of the proportion of differentiated thyroid cancers, nodule size >1 cm and nodule size ≤1 cm in male and female during 2000~2013. (**A**) The proportion of differentiated thyroid cancers; (**B**) the proportion of nodule size >1 cm; (**C**) the proportion of nodule size >1 cm.

As shown in Fig. 2A, the proportion of DTC increased during in the patients with thyroidectomies both in males and in females. The proportion increased along with the increase of year during 2000–2013, which was from 7.5% to 68.1% in males (P < 0.05) and from 16.2% to 66.7% in females. While the proportion of nodule size > 1.0 cm decreased during this period whether in males or in females (Fig. 2B). Of course, it was opposite to the proportion of nodule size ≤ 1.0 cm (Fig. 2C).

### BMI

**Figure 3:**
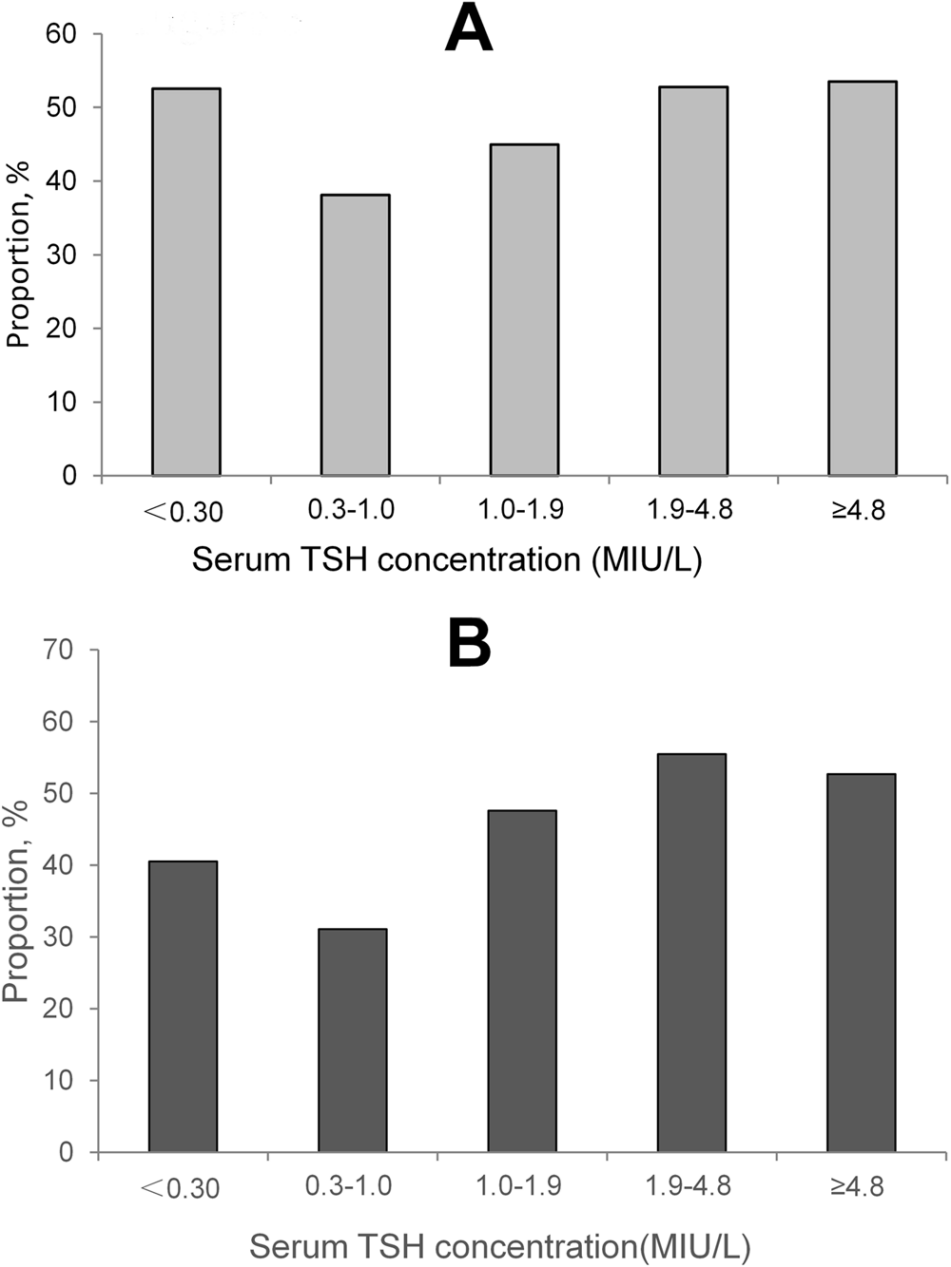
The distribution of serum TSH concentration in the patients with differentiated thyroid cancers in female and male. (**A**) female; (**B**) male.

The differences of proportion of DTC were no significant among the four BMI groups in female, while the U shape was observed in male (Fig. 3). Compared to the female, in the obesity groups, the proportions of DTC in male were higher in the obesity group (P = 0.001) and lower in the normal group (P < 0.001).

### TSH

The level of preoperative TSH was significantly higher in patients with DTC compared to the patients with benign (1.97 vs. 1.57 mIU/L, P < 0.001), which was similar in male (1.66 vs. 1.44 mIU/L, P = 0.001) and in female (2.08 vs. 1.62 mIU/L, P < 0.001) in females (Table 1), respectively.

The proportions of DTC in male (38.1%) and in female (31.1%) were lowest in in the 0.3–1.0 MIU/L group of serum TSH (Fig. 3). There were no significant differences of serum TSH between male and female in the four groups (all P > 0.05). The proportion of DTC was highest (55.5%) in the 1.9–4.8 MIU/L group of serum TSH in female, while it was highest in the >4.8 MIU/L group in male.

### Blood types

**Figure 4:**
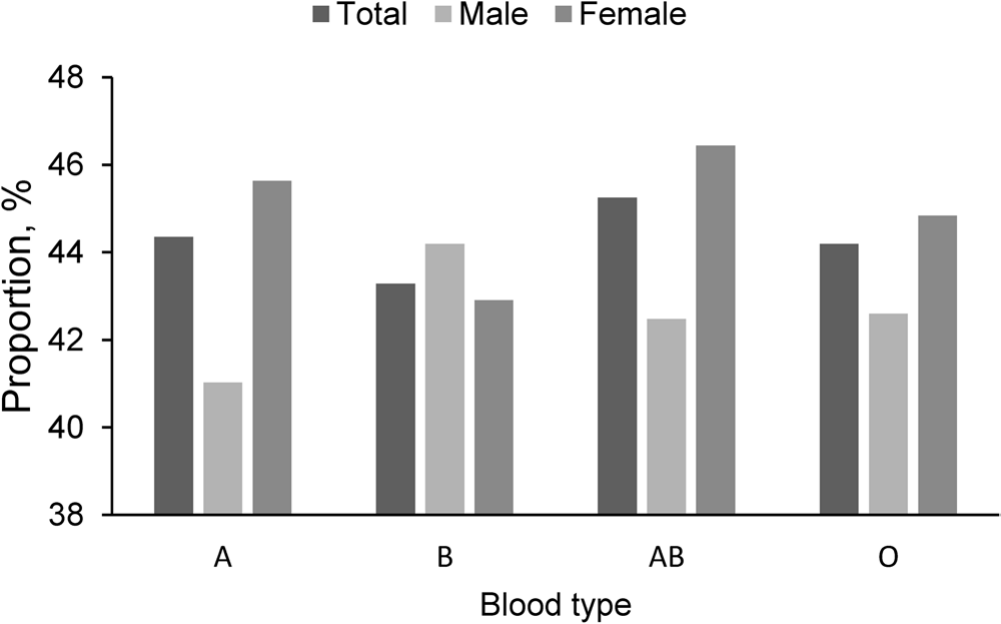
The distribution of the proportion of differentiated thyroid cancers in different blood type in male and female.

Of all the thyroid nodules, the percentage of thyroid cancer in thyroid nodules was higher in patients with blood type AB than other blood types (Fig. 4). The similar result was observed in female and the lowest proportion was blood type B. However the proportion of thyroid cancer was dominated in blood type B and the lowest incidence in blood type A in male. But there were no significant difference in different blood type.

### Multiple analysis

The results of logistic regression model showed that age, nodule number, BMI and serum TSH were the related factors for DTC in the study subjects (Table 3). Compared with normal weight group, the OR of the thin, partial fat and obesity for DTC were 1.304 (95%CI 1.064–1.598), 046 (95%CI 0.03–0.73) and 1.22 (95%CI 1.043–1.425) However, when male and female were analyzed separately, compared with the normal weight group, only OR of the thin was statistically significant. The OR for DTC increased along with serum TSH increasing when serum TSH < 4.8 mIU/L. Higher TSH level was related to the DTC occurrence in male or female. However, compared to the group of 0.3 ≤ TSH < 1.0 mUI/L, the patients with TSH > 0.3 mUI/L or 1.0 ≤ TSH < 1.9 mUI/L had a significant higher proportion in female. The patients with multiple nodules had a higher proportion of DTC compared to single nodule (OR = 1.185, 95%CI 1.015–1.383, P = 0.031) while no significant differences were found in male or female.

**Table 3:** The related factors of thyroid malignancy by logistic regression model.

Variables	OR	95%CI		P value
		Lower	Upper	
**Overall**				
Gender	0.966	0.823	1.132	0.666
Nodule number (single)	1.185	1.015	1.383	0.031
Age	0.954	0.948	0.960	<0.001
**Body mass index (kg/m** ^**2**^ **)**				
18.5–24	1.000			
<18.5	1.304	1.064	1.598	0.011
24–28	0.462	0.294	0.728	0.001
≥28	1.221	1.043	1.425	0.013
**Thyroid stimulating hormone**				
<0.3	1.000			
0.3–1.0	1.730	1.395	2.146	<0.001
1.0–1.9	2.465	2.000	3.037	<0.001
1.9–4.8	2.566	1.917	3.434	<0.001
≥4.8	1.527	1.122	2.080	0.007
**Male patients**				
Age	0.934	0.922	0.946	<0.001
Nodule number (single)	1.048	0.786	1.397	0.751
**Body mass index (kg/m** ^**2**^ **)**				
18.5–24	1.000			
<18.5	1.632	1.122	2.376	0.010
24–28	0.956	0.183	5.000	0.958
≥28	1.342	0.959	1.879	0.086
**Thyroid stimulating hormone**				
<0.3	1.755	0.938	3.284	0.078
0.3–1.0	1.000			
1.0–1.9	1.235	0.851	1.792	0.267
1.9–4.8	1.780	1.219	2.599	0.003
≥4.8	2.649	1.395	5.030	0.003
**Female patients**				
Age	0.964	0.956	0.971	<0.001
Nodule number (single)	1.208	1.001	1.447	0.039
**Body mass index (kg/m** ^**2**^ **)**				
18.5–24	1.000			
<18.5	0.444	0.275	0.716	0.001
24–28	1.164	0.972	1.395	0.099
≥28	1.123	0.867	1.454	0.380
**Thyroid stimulating hormone**				
<0.3	1.441	1.002	2.071	0.049
0.3–1.0	1.000			
1.0–1.9	2.058	1.574	2.691	<0.001
1.9–4.8	2.824	2.188	3.645	<0.001
≥4.8	2.644	1.890	3.699	<0.001

### Pathological characteristics of DTC

Compared with female, male had significantly higher rates of III/IV TNM Stage (12.3% vs. 18.7%, P < 0.001), lymph node metastasis (24.1% vs. 32.9%, P < 0.001) and extra thyroidal invasion (3.7% vs. 7.0%, P < 0.001) in Table 4.

**Table 4:** Clinical pathological characteristics of DTC.

Factors	Male (n, %)	Female (n, %)	P Value
TNM Stage			0.001
I/II	743(81.8)	2025(87.66)	
III/IV	165(18.2)	285(12.34)	
Lymph node metastasis			<0.001
No	609(67.1)	1752(75.9)	
Yes	298(32.9)	555(24.1)	
Extra thyroidal invasion			<0.001
No	842(93.0)	2220(96.4)	
Yes	63(7.0)	84(3.65)	

## Discussion

Thyroid cancer is the most common endocrine malignancy with the increasing new patients diagnosed worldwide. In the United States, thyroid cancer has been reported to account for about 1.0–1.5% of newly diagnosed cancer^1^. The tumor of thyroid has long been established to have an obvious female predominance. It has been ranked fifth in the most common cancer of female, while not in the list of 15 most common male cancers^13^. However, male patients with thyroid cancer had poor prognosis than their female counterparts^7^. The study by Li *et al*. has indicated that gender was an independent predictive factor for malignant thyroid nodules, with male patients had 0.78-fold risk for development of malignant thyroid nodules^6^. This study showed that tumor sizes of our male patients were larger than that of female patients. There were more patients of advanced thyroid cancer, lymphatic metastasis and extra-thyroid invasiveness in male patients, suggested the more malignant behaviors in male patients with thyroid cancer.

According to the investigation by Yang *et al*., the standardized incidence rates of thyroid cancer in urban Beijing were 11.1% in male and 12.1% in female during 1995 to 2010^14^. The development of thyroid cancer has been suggested to associate with age and gender of the individuals. In the present study, we mainly focused on the thyroid cancer patients underwent thyroidectomy. In thyroid nodule patients underwent thyroidectomy, the incidence of thyroid cancer showed the similar annual increase rate between male and female patients based on the hospital data. These results were consistent with the previous study, which indicated that, despite of female predominance for thyroid cancer, female thyroid nodule patients that underwent thyroidectomy had no significant high risk for development of thyroid cancer than their male counterparts.

The inherited human blood group antigens have been previously indicated to associate with the risk of various malignancies^9,10^. Human blood antigens are glycoproteins expressed on the surface of red blood cells and a few other cell types, including cells from the gastrointestinal tract. The sugar residues of these glycoproteins may attach to the H antigen, a protein backbone, by ABO gene-encoded glycosyltransferase. Alterations of surface glycoconjugates may lead to modifications that related to the tumor development and spread. One study by Gong *et al*. in China indicated that individuals with blood type B were more likely to develop esophagus cancer and cholangiocarcinoma^8^. Our study showed that the risk of thyroid cancer was significantly higher in blood type B patients (OR = 1.30, 95% CI = 1.02–1.66) compared with patients with other blood types.

The findings of our study showed that the prevalence of thyroid cancer was higher among male thyroid nodule patients with blood type B when compared with these with other blood types. By contrast, in the female counterpart, thyroid nodule patients with blood type AB showed higher risk for development of thyroid cancer. However, the data were not found to be statistically significant.

The incidence of thyroid cancer has been reported to be comparatively higher and occurs earlier in female patients than their male counterparts, with a male/female ratio of 1:3. However, there was no gender predilection in the onset of thyroid cancer in our study. The age of DTC diagnosis was not significantly different between male and female patients, while other clinical characteristics were found to be markedly different.

Previous meta-analysis showed that patients with multi-nodular goiters have lower risk for development of thyroid cancer than these with uninodular goiter^15^. Fine needle aspiration has been recommended in uninodular goiter patients with tumor size of greater than 1 cm according to the treatment guidelines of thyroid nodule; while in patients with multi-nodular goiters, fine needle aspiration was recommended when the clinical symptom or ultrasound examination indicated malignant disease^6,16^. Corroborating the results of previous study, present study showed that the incidence of DTC was obviously higher in patients with uninodular goiter than that of multi-nodular goiter patients. Further gender-based analysis indicated that, in male, uninodular goiter patients have a higher risk of development of DTC than their multi-nodular goiter counterparts. By contrast, no significant difference was observed in female patients with uni- or multi-nodular goiters. These results indicated that male and uninodular goiter may be the independent risk factors for development of DTC, and patients with male gender and uninodular goiter should be clinically managed more cautiously.

Despite the lower incidence of male thyroid cancer, the disease has been reported to be more invasive in male patients, with the higher lymphatic metastasis and poorer prognosis^17^. Our study showed more cases of male patients with advanced DTC, and the incidence of lymphatic metastasis and extra-thyroid invasiveness were significantly higher in male DTC patients when compared with female patients. This gender-related difference may be partially explained by the younger age (<55 years) of the DTC patients recruited in this study. Previous study has indicated that, female patients with thyroid cancer diagnosed before 55 years of age had better prognosis than their male counterparts. However, no prognostic difference was observed between male and female patients when the disease was diagnosed later than 55 years old. Estrogen and estrogen receptor may play a critical role in the gender-related difference of patients younger than 55 years of age^18^.

The present study described the gender-related difference in clinical and ultrasound characteristics, tumor staging, and aggressive behavior (lymphatic metastasis and extra-thyroid invasiveness) of the DTC patients. Factors, including diet, environment, hormone, heredity, genetic variation, and etc., may all play a role in this procedure. Our future study may focus on the potential mechanisms of this gender-related difference in thyroid cancer, and genome-wide single nucleotide polymorphism analysis may be used to identify novel susceptibility genes. Besides, in view of the more aggressive behaviors of DTC in male patients, much more attention should be focused on the timely diagnosis and treatment of these patients.
